# Development of a Topical Insulin Polymeric Nanoformulation for Skin Burn Regeneration: An Experimental Approach

**DOI:** 10.3390/ijms22084087

**Published:** 2021-04-15

**Authors:** Maria Quitério, Sandra Simões, Andreia Ascenso, Manuela Carvalheiro, Ana Paula Leandro, Isabel Correia, Ana Silveira Viana, Pedro Faísca, Lia Ascensão, Jesús Molpeceres, Maria Manuela Gaspar, Catarina Pinto Reis

**Affiliations:** 1 iMed., ULisboa, Research Institute for Medicines, Faculty of Pharmacy, Universidade de Lisboa, 1649-003 Lisboa, Portugal; mariaquiterio@campus.ul.pt (M.Q.); ssimoes@ff.ulisboa.pt (S.S.); andreiaascenso@ff.ulisboa.pt (A.A.); mcarvalheiro@ff.ulisboa.pt (M.C.); aleandro@ff.ulisboa.pt (A.P.L.); 2 Centro de Química Estrutural, Instituto Superior Técnico, Departamento de Engenharia Química, Universidade de Lisboa, 1049-001 Lisboa, Portugal; icorreia@tecnico.ulisboa.pt; 3 Centro de Química Estrutural, Faculdade de Ciências, Universidade de Lisboa, 1749-016 Lisboa, Portugal; apsemedo@fc.ul.pt; 4 Faculdade de Medicina Veterinária, Universidade Lusófona de Humanidades e Tecnologias, 1794-024 Lisboa, Portugal; pedrofaisca@ulusofona.pt; 5 CBIOS-Research Center for Biosciences & Health Technologies, Universidade Lusófona de Humanidades e Tecnologias, 1794-024 Lisboa, Portugal; 6 Centro de Estudos do Ambiente e do Mar (CESAM), Faculdade de Ciências, Universidade de Lisboa, 1749-016 Lisboa, Portugal; lmpsousa@fc.ul.pt; 7 Department of Biomedical Sciences, Faculty of Pharmacy, University of Alcalá, Campus Universitario, 28871 Alcalá de Henares, Spain; jesus.molpeceres@uah.es; 8 IBEB, Biophysics and Biomedical Engineering, Faculdade de Ciências, Universidade de Lisboa, 1749-016 Lisboa, Portugal

**Keywords:** wound healing, insulin, nanoparticles, PLGA, topical administration

## Abstract

Insulin is a peptide hormone with many physiological functions, besides its use in diabetes treatment. An important role of insulin is related to the wound healing process—however, insulin itself is too sensitive to the external environment requiring the protective of a nanocarrier. Polymer-based nanoparticles can protect, deliver, and retain the protein in the target area. This study aims to produce and characterize a topical treatment for wound healing consisting of insulin-loaded poly-DL-lactide/glycolide (PLGA) nanoparticles. Insulin-loaded nanoparticles present a mean size of approximately 500 nm and neutral surface charge. Spherical shaped nanoparticles are observed by scanning electron microscopy and confirmed by atomic force microscopy. SDS-PAGE and circular dichroism analysis demonstrated that insulin preserved its integrity and secondary structure after the encapsulation process. In vitro release studies suggested a controlled release profile. Safety of the formulation was confirmed using cell lines, and cell viability was concentration and time-dependent. Preliminary safety in vivo assays also revealed promising results.

## 1. Introduction

Skin burns are one of the most frequent cutaneous lesions with several causes, such as: excessive exposure to a source of heat, caustic chemicals, exaggerated sun exposure, and radiation [1]. Burns can be classified based on the extension and depth of the lesion, changing the healing time of the skin [2]. Wounds can be clinically categorized as acute or chronic according to their time frame of healing. Acute wounds can be formed due to traumatic loss of tissue or a surgical procedure, whereas chronic wounds are those that fail to progress through the normal stages of healing and cannot be repaired in an orderly and timely manner [3].

Most burns are treated using gauze dressings to isolate the wound from the external environment, albeit not having the inherent capacity to promote wound healing, and possibly destroying the newly formed tissue layer at the time of dressing removal [4]. The potential use of growth factors has been investigated [5,6]. Nevertheless, these agents might present some disadvantages, such as short stability, high cost and usually difficult manipulation [7].

Insulin is a peptide and a growth factor with several physiological roles. The role of insulin in the skin is not a new subject, since previous studies confirmed the presence of insulin receptors in the keratinocytes and fibroblasts of the epidermis. Human keratinocytes are insulin-dependent for their growth. Moreover, insulin can act as a chemoattractant and mitogenic agent for cells that are involved in healing [8,9,10]. It has been stated that insulin increases protein synthesis in the skin, and stimulates the growth and development of different cell types affecting the proliferation, migration, and secretion of keratinocytes, endothelial cells, and fibroblasts with a known pivotal role in the development of granulation tissue [9]. Comparing to other potential therapeutic agents, insulin is quite interesting for burn and wound care [11] with the additional benefit of being a low-cost approach.

As a protein, insulin is sensitive to several external factors, such as temperature, pressure, and radiation. On the other hand, it has been described that conventional wound healing drugs have limited skin penetration, a limitation that can be overcome through drug loading in nano-DDs (drug delivery systems) [12]. These systems offer several advantages over the micro- or larger DDs, and they are obtained from natural, semi-synthetic or synthetic materials. The nature of the used material will define their biocompatibility. In general, most nano-DDs are found to be compatible with skin [13,14,15]. Poly (lactic-co-glycolic acid) (PLGA) is one of the most widely used polymers to prepare nanoparticles because it is biocompatible and biodegradable [16]. It is expected that the hydrophobic polymer, PLGA, will release hydrophilic insulin after topical application. Then, PLGA nanoparticles will undergo hydrolysis, breaking its ester linkage to form lactic acid and glycolic acid monomers that can be easily metabolized [17]. In addition, according to previous studies, the released lactate also plays a role in the treatment of wounds by accelerating angiogenesis and the wound healing process [18].

The current study focuses on the preparation and characterization of insulin-loaded PLGA (nanoparticles) NPs in terms of size and polydispersity index (PdI) by dynamic light scattering (DLS); NPs surface charge by electrophoretic mobility; morphology by scanning electron microscopy (SEM) and atomic force microscopy (AFM); encapsulation efficiency by HPLC; insulin structural preservation by far-UV circular dichroism (far-UV CD) and denaturant polyacrylamide gel electrophoresis (SDS-PAGE) to evaluate insulin structural integrity after the encapsulation process. Formulation stability studies were also carried out over 60 days. In vitro release and diffusion assays were performed to characterize the release profile of insulin from the topical formulation. Safety was assessed by in vitro assays using appropriate cellular models and in vivo approaches using animal models.

## 2. Materials and Methods

### 2.1. Materials

#### 2.1.1. Chemicals

Insulin (100 I.U./mL) was obtained from a commercial insulin solution for injection. PURASORB^®^ PDLG 5002–PLGA Ratio L/G% 50:50 (MW 45,000–7500) was purchased from PURAC (AA Gorichem, The Netherlands). Pluronic ^®^ F127 (POLX) was acquired from Sigma-Aldrich ™ (St. Louis, MO, USA). MiliQ Water by Millipore Corporation (Burlington, MA, USA). All the chemical products and solvents used were of analytic purity grade.

#### 2.1.2. Immortalized Human Keratinocytes Cells (HaCat)

The HaCaT cell line was obtained from Cell Lines Services (CLS) (Eppelheim, Germany). Cells were aseptically grown in RPMI1640^®^ high glucose culture medium supplemented with 10% fetal bovine serum (FBS), 1% penicillin-streptomycin (10,000 U/mL, Gibco, Life Technologies, Granad Island, NY, USA) at 37 °C in a humidified atmosphere with 5% CO_2_.

#### 2.1.3. Animals

Eight-week-old female CD-1 mice (25–40 g) were purchased from Charles River (Barcelona, Spain). The animal housing was kept at the controlled temperature of 22.0 ± 1.0 °C, humidity at 50 ± 15%, and a light/dark cycle of 12 h. Animals were kept under standard hygiene conditions, fed with commercial chow, and given acidified drinking water ad libitum.

All animal experiments were conducted in accordance with the EU Directive (2010/63/UE), the Portuguese law (DR 113/2013, 2880/2015, 260/2016 and 1/2019), and the Animal Welfare Commission of the Faculty of Pharmacy, Universidade de Lisboa and by the competent national authority *Direção-Geral de Alimentação e Veterinária* (DGAV).

### 2.2. Methods

#### 2.2.1. Preparation of NPs

Insulin-loaded NPs were prepared using a previously modified-spontaneous emulsification solvent diffusion method [19,20]. Briefly, a specific amount of insulin and PLGA were added to 5 mL of the organic solution. The organic solution was prepared using ethanol and acetone (2:8, *v*/*v*). PLGA and insulin suspension were slowly added to 10 mL of POLX (1%, *m*/*v*), under a constant stirring of 800 rpm (Heidolph MR 3001, Heidolph Instruments, Schwabach, Germany) for 15 min at room temperature [20].

The NPs’ suspension was immediately formed and named as insulin-loaded NPs. Then, half of this suspension was evaporated under reduced pressure (RV 10 digital from VWR by IKA) to eliminate organic solvents. The other half was not evaporated. Both samples were then stored at 4 °C for further analysis. Empty NPs (without insulin) were prepared using the same method.

#### 2.2.2. Preparation POLX-Based Hydrogel

POLX was used as gelling agent to avoid any different material besides the materials used in NPs preparation. Moreover, this hydrogel has additional interesting properties that could help retain the formulation at the target area. The so-called “cold-method” was adopted to prepare the gel as described by Soga et al. [21]. Briefly, POLX (1.8 g) was added to 10 mL of PBS (pH 7.4, USP 39) aqueous solution in a glass vial, and gently mixed with magnetic stirring for 24 h at 4 °C until all the POLX granules were completely dissolved, and a clear solution was obtained.

#### 2.2.3. NPs Characterization

Mean size, Polydispersity Index (PdI), Zeta Potential, and pH analysis.

Particle size and polydispersity index (PdI) were measured by DLS using a Zetasizer Nano-Z Malvern (Malvern, Worcestershire, UK) instrument at a constant temperature of 25 °C and detection angle of 165°. Samples were diluted in water for DLS analysis (dilution factor of 1:10).

Surface charge was analyzed by electrophoretic mobility technique using a Zetasizer Nano-Z Malvern equipment (Malvern, Worcesterchire, UK). The samples were diluted in 0.1M of NaCl (dilution factor of 1:50). Each sample was tested in triplicate for both techniques.

pH measurement of insulin-loaded NPs suspension was performed in triplicate using a pH 50^+^ DHS pHmeter (XS instruments, Rome, Italy).

#### 2.2.4. Surface and Morphological Analysis

Particle morphology was observed by AFM and SEM. AFM analysis was carried out using a Multimode 8 HR microscope coupled to a Nanoscope V Controller (Bruker, UK) and a Peak Force Tapping mode, under ScanAsyst control. The probe model used was ScanAsyst-air with a spring constant of 0.4 N/m (Bruker). For AFM imaging, a drop of ca. 30 µL of both empty and insulin-loaded NPs was placed on a freshly cleaved mica surface and air-dried.

For SEM, aliquots (10 µL) of aqueous suspensions containing NPs (empty and loaded) were carefully scattered over a round glass coverslip previously attached to the microscope stubs. Afterwards, samples were left to dry in a desiccator, being subsequently coated with a thin layer of gold and observed on a JEOL 5200LV scanning electron microscope (JEOL Ltd., Tokyo, Japan) at an accelerating voltage of 20 kV.

In both techniques (AFM and SEM), images were recorded digitally.

#### 2.2.5. Determination of Encapsulation Efficiency (EE)

The percentage of insulin encapsulated was indirectly determined by quantifying the amount of insulin present in the supernatant after centrifugation of samples (10,000× *g*, 15 min, using a Beckman Instrument centrifuge, Inc., Brea, CA, USA). For insulin quantification, an HPLC system was used (Hitachi System LaCrom Elite, Column oven, Diode Array Detector UV-VIS and Pump, Tokyo, Japan). Separation was performed on a Waters Symmetry C18, 5 µm column (4.6 × 150 mm) with an isocratic flow of 0.7 mL/min. The mobile phase was composed of acetonitrile:water with TFA (60:40; *v*/*v*). Using the described conditions, a retention time of 3.1 min was obtained for insulin. The measurements were performed in triplicate, and the calibration was done with a standardized solution of insulin, at λ 220 nm. The linearity range was established in the 1.09–70 µg/mL range the detection limit was 0.358 µg/mL, and the quantification limit was 1.087 µg/mL. Encapsulation efficiency (EE) was then determined by using Equation (1):

(1) $$EE\left(\%\right)=\frac{\left(Initially\;added\;insulin-insulin\;present\;in\;supernatant\right)}{Initially\;added\;insulin\;}\times 100$$

#### 2.2.6. Physical Stability of Insulin Nanoformulation over Time

Stability studies were performed in three independent batches of insulin-loaded NPs stored at 4 °C, over 60 days. The formulation parameters (size, PI, zeta potential, pH, and drug loading over time) were determined in each predetermined period, using the same techniques and methodologies as described before.

#### 2.2.7. Protein Structural Stability after Encapsulation

##### SDS-PAGE

For analysis of peptide integrity, 20 µL of samples containing 18.6, 11.6, and 5.8 µg of insulin were loaded into a Bis-Tris 15% gel (Invitrogen, Thermo Fisher Scientific). Before loading, samples were mixed with 4.0 µL of loading buffer (62.5 mM Tris-HCl, pH 6.8; 2% SDS, 0.025% Bromophenol blue, 20% Glycerol, 5% β-mercaptoethanol) and heated at 100 °C, for 10 min. Electrophoretic separation was performed on a mini-PROTEAN^®^ Tetra System (Bio-Rad), at 50 mV, using the running buffer (62.5 mM Tris, 192 mM Glycine, 0.1% SDS, pH 8.3), until the reference dye (Bromophenol blue) reached the bottom of the gel. The BlueSafe dye (Nzytech, MB 15201) was used to visualize the denatured protein bands.

##### Circular Dichroism

The secondary structure of insulin released from NPs was analyzed by far-UV CD spectroscopy. Spectra were recorded on a JASCO J-720 spectropolarimeter (JASCO, Hiroshima, Japan) equipped with a temperature controller. The temperature was kept constant at 25 ± 1 °C, and spectra were measured from 200–300 nm. The samples for far-UV CD analysis were obtained by dissolution of the NPs in PBS buffer, and insulin concentrations were normalized to 0.41 mg/mL. The spectral signatures of the samples were compared to the spectrum of commercial insulin (free insulin) at the same concentration.

#### 2.2.8. Release Behavior of Insulin Formulations over Time

In vitro release assay was carried out using Float-a-Lyzer (100 KD MWCO, 5-mL capacity, regenerated cellulose membrane) that was purchased from Spectrum Labs (Rancho Dominguez, CA, USA). The 100 KD MWCO membrane selected was sufficiently large to allow passage of the three forms of the insulin protein (monomer, dimer, and hexameric forms). About 5 mL of insulin-loaded NPs suspension was transferred to the dialyzer. The dialyzer was then introduced into a 25-mL glass cylinder containing PBS at pH 7.4 (USP 39) as a release medium as recommended by OECD guideline 428, which was stirred at 1000 rpm using a magnetic stir bar. Insulin release was assessed by intermittently sampling the contents (250 µL) of the outer medium. Replacement of the same amount of fresh buffer occurred immediately after sampling to maintain the sink conditions.

A simultaneous assay using only insulin solution was performed as a control. A solution of commercial insulin at 90 mg/mL was diluted in 1 mL of PBS, at 37 °C with a constant stirring of 100 rpm for 127 h.

The insulin released in analyzed samples was determined using the previously described HPLC method.

##### In Vitro Diffusion Assay Using Franz Cells

In vitro diffusion assay was carried out using Franz cells and silicone membranes. Each cell consists of two chambers, the donor and the receptor compartments separated by the synthetic membrane with a diffusion area of 1.00 cm^2^. The donor compartment was filled with 0.5 mL (infinite dose) of insulin-loaded NPs formulation or commercial insulin solution and covered with parafilm. PBS at pH 7.4 (USP 39) was used as a receptor medium as recommended by OECD guideline 428. The Franz cells were placed over a magnetic stirrer, and the bath temperature was maintained at 32–37 °C.

Then, 0.5 mL of medium was collected from the receptor compartment at predetermined intervals of 1 h over 8 h, being replaced with the same amount of fresh buffer to maintain the sink conditions.

Insulin was quantified in collected samples using the previously described HPLC method.

#### 2.2.9. Preliminary In Vitro Safety Assessment

##### In Vitro Assessment

The safety of insulin-loaded NPs was in vitro assessed using 3-(4,5-dimethylthiazol-2-yl)-2,5-diphenyltetrazolium bromide (MTT) assay (Sigma, St. Louis, MO, USA) [22]. Considering the topical administration of insulin-loaded NPs, an adherent immortalized human keratinocytes non-tumorigenic cell line (HaCaT) was used for the cell viability assay. Harvested cells from exponential-phase cultures were seeded in 96-well plates (20,000 cells/well) and incubated for 24 h at 37 °C in a humidified CO_2_ atmosphere to allow cells to adhere.

Then, cells were exposed to a concentration range of 2.5 µg/mL to 80 µg/mL of insulin-loaded NPs in POLX gel, commercial (free) insulin in POLX gel, and empty PLGA NPs in POLX gel (equivalent concentration) for 12 h and 24 h.

After the exposure period, the medium was carefully removed, and cells were washed once with 200 µL/well of PBS. Then, 50 µL of an MTT solution (0.5 mg/mL) was added to each well and left to incubate for 4 h in the tissue incubator. Afterward, 200 µL of DMSO (Sigma, St. Louis, MO, USA) was added per well to solubilize the formazan purple crystals, and the plates were gently stirred for 15 min. The optical density of reduced MTT was measured at 570 nm by spectroscopy on an automatic microtiter plate reader (Model Elx 800, Biotek, Winooski, VT, USA).

The percentage of cell viability was calculated by the following Equation (2):

(2) $$Cell\;viability=\frac{Abs\;samples}{Abs\;control}\times 100$$

where *Abs samples* is the absorbance of cells incubated with the tested formulations and *Abs control* is the absorbance of the cells incubated with the culture medium.

#### 2.2.10. Preliminary In Vivo Efficacy Assessment

To assess the formulation efficacy, preliminary in vivo assays were performed in CD-1 mice. The animals were randomly allocated into the experimental groups: group 1 with animals treated with the vehicle of the test formulation (only POLX gel) (n = 3); group 2 with animals treated with a commercial cream of silver sulfadiazine at the concentration of 10 mg/g of cream (n = 3); group 3 with animals treated with commercial insulin solution (n = 4) and group 4 with animals treated with insulin-loaded NPs in POLX gel (n = 4).

**Figure 1:**
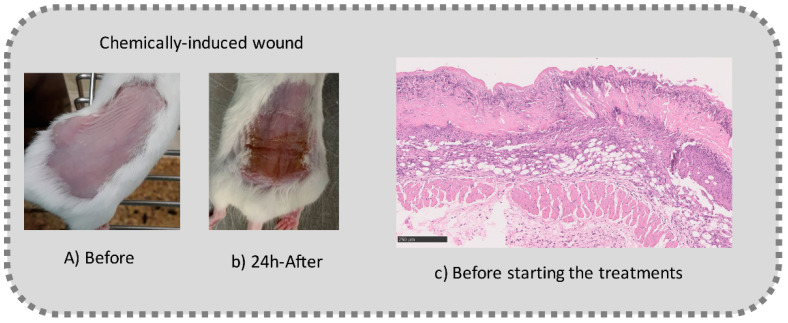
Skin aspect and wound creation: (**A**) Before and (**b**) 24 h-after chemical induction, and (**c**) histological analysis of skin damage before starting the treatment (magnification of 40 ×, scale bar 250 µm).

Before the beginning of the experimentation, an area of a minimum of 2 cm^2^ on the back of each mouse was shaved with a commercial depilatory cream to expose the skin. Then, animals were lightly anesthetized with isoflurane and the chemical burn was induced by topical application of 100 µL of Texapon^®^ N 70 (Sodium Laureth Sulfate, BASF SE, Germany) for two consecutive days. Twenty-four hours after, a skin burn rapidly appeared, as seen in Figure 1. Histological analysis (before treatments) showed the impact of this chemical burn in the skin’s layers.

To avoid any pain or discomfort, all topical treatments were performed after light sedation with isoflurane. Formulations were applied topically (100 µL) on the top of the lesions for five consecutive days. Throughout the model time course, all animals had food and water ad libitum. The water provided during the experiment contained codeine (30 mg/500 mL) also to ensure the animals’ welfare.

During the 8-days experiment, the evolution of the burns was recorded by a daily photograph and by measuring the skin thickness using a caliper.

On the last day, specimens of skin were excised and fixed in 10% buffered formalin for a minimum period of 48 h. Then, they were routinely processed, embedded, sectioned into 5-μm-thick sections, and stained with H&E. Slides were analyzed with a BX31 microscope (Olympus Corporation, Tokyo, Japan), and images were acquired with the NanoZoomer-SQ Digital slide scanner C13140-01 (Hamamatsu Photonics, Shizuoka, Japan).

A scoring system for wound healing was developed by adaptation of previously published scoring systems [23,24]. Briefly, skins were scored for epidermal closure (ulcer-0 or closed-1), epidermal differentiation (absent-0, spinous epidermal-1 or granular layer-2), amount of granulation tissue (profound-1, moderate-2, scanty-3 or absent-4), inflammatory infiltrate (plenty-1, moderate-2 or few-3), collagen fiber orientation (vertical-1, mixed-2 or horizontal-3) and pattern of collagen (reticular-1, mixed-2 or fascicle-3).

#### 2.2.11. Statistical Analysis

The results are reported as mean ± standard deviation (SD) of at least three replicates. The results were statistically analyzed by *t*-test or by analysis of variance (ANOVA) for comparison between two or more groups, respectively, using SigmaPlot 11.0 software (Slough, UK). The overall significance level *p* is 0.05.

## 3. Results

### 3.1. NPs Characterization: Size, PI, Zeta Potential, Particle Morphology, and EE

**Figure 2:**
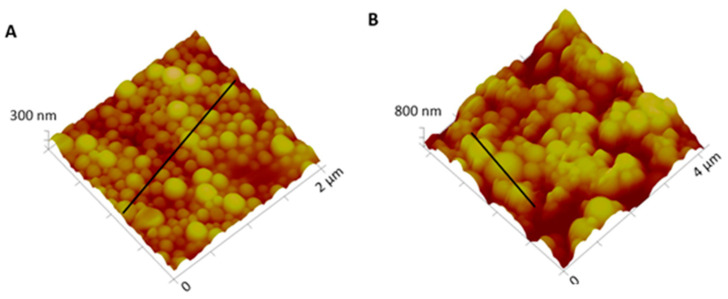
Three-dimensional atomic force microscopy (AFM) topography images and corresponding profiles: (**A**) Empty NPs and (**B**) insulin-loaded NPs.

**Figure 3:**
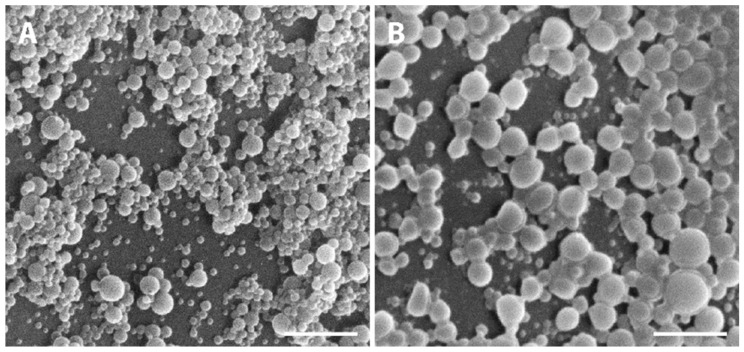
Representative SEM micrographs showing the PLGA NPs. (**A**) Empty PLGA NPs and (**B**) insulin-loaded NPs. Scale bars correspond to 3 µm.

Macroscopically, NPs suspension presented a whitish macroscopic appearance. Particle morphology and shape were characterized by AFM and SEM techniques. Figure 2 shows AFM images and corresponding profiles of both empty and insulin-loaded NPs, and Figure 3 displays SEM analysis of the same batch of NPs. By AFM and SEM individualized NPs, with well-defined spherical shapes and smooth surfaces can be depicted in both samples. The sizes observed for loaded NPs range from 300–600 nm (Figure 2B), which are considerably larger than those obtained for empty NPs, typically between 100 and 260 nm (Figure 2A). The change in size was also clearly observed in SEM images (Figure 3A,B).

Table 1 summarizes the main results of the NPs characterization. As observed by DLS analysis, empty PLGA NPs presented a mean size around 220 nm, and after insulin loading, the particle size increased up to 570 nm, corroborating AFM and SEM results. This change was probably due to the size of insulin. Concerning PdI, values were lower than 0.4, indicating that this suspension had a very low tendency to aggregate [25], a fact that was further confirmed by stability studies.

The surface charge, assessed by zeta potential measurement, showed that empty and insulin-loaded polymeric NPs was neutral. The encapsulation of insulin increased the formulation pH; however, the values are still suitable for topical application. Encapsulation efficiency, another extremely important parameter in drug-NP characterization, was 55.3 ± 0.01% for insulin-loaded NPs.

According to Table 1, the values of the evaluated parameters were similar for both evaporated and non-evaporated insulin-loaded NPs, so the evaporation process had no impact on the parameter’s outcome. Although the number of residual solvents was not monitored in this work, considering that both solvents utilized are class III (ICH guideline Q3C (R7) on impurities: Guideline for residual solvents), the potential concern related to the application of this formulation is quite small. Moreover, this formulation is intended for acute situations. Thus, chronic exposure will be less probable. Therefore, for the following tests, the non-evaporated formulation was used.

### 3.2. Physical Stability of Nanoformulation over Time

**Figure 4:**
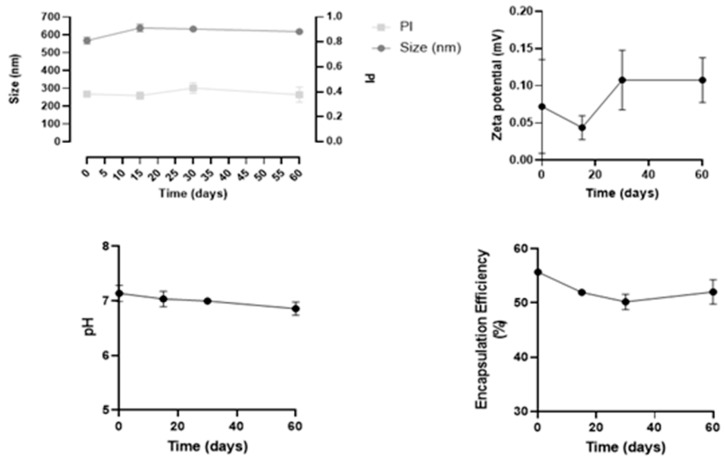
Results of stability studies of insulin-loaded NPs over 60 days. All data are presented as mean ± SD (n = 3).

Stability studies were performed in three independent batches of insulin-loaded NPs, over 60 days regarding the following parameters: size, PdI, zeta potential, pH, and drug loading over time, as shown in Figure 4.

Overall, this formulation revealed to be stable for at least 60 days, since both physical and chemical parameters remained constant, and no statistical differences were observed over time. In particular, the mean size remained 543 ± 7 nm, and PdI values were between 0.3 and 0.4. These values are considered acceptable and commonly found for NPs, as reported in the literature [26].

### 3.3. Protein Stability after Encapsulation Process

**Figure 5:**
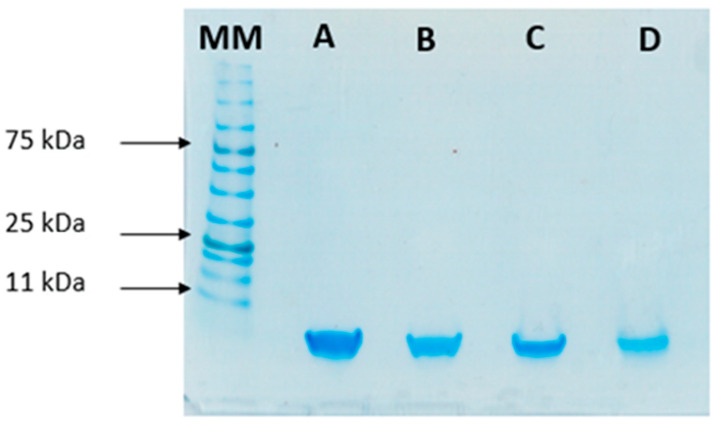
SDS-PAGE analysis of insulin: (**A**) Free insulin (commercial insulin); (**B**) insulin present in total formulation (encapsulated and non-encapsulated insulin); (**C**) insulin in the supernatant after centrifugation of NPs’ suspension; and (**D**) insulin released from the NPs. MM, molecular mass marker (NzyColour protein marker II, Nzytech).

To study the stability of insulin during the formulation process, SDS-PAGE was performed to compare the migration pattern of insulin (5.8 kDa) before and after encapsulation. This analysis was used as an indicator of the protein’s integrity after encapsulation. Bands in Figure 5 show that both standard insulin and insulin released from loaded NPs have a similar molecular mass of 6 kDa, confirming its structural integrity.

**Figure 6:**
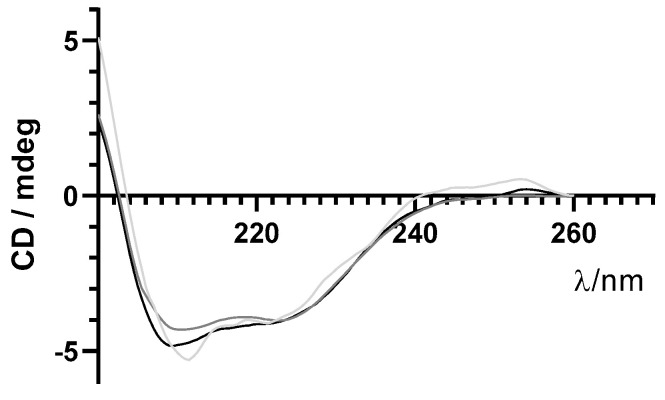
Far−UV circular dichroism (CD) spectra of free insulin (grey line), insulin-loaded NPs (light grey line), and supernatant (darker grey line) in the far−UV range.

As previously mentioned, maintenance of structural integrity of insulin during encapsulation in PLGA NPs is crucial for its therapeutic effect. Far-UV CD spectroscopy was used to monitor the secondary structure of insulin before and after the encapsulation process. Similar far-UV CD spectra of insulin released from NPs and commercial-free insulin are shown in Figure 6. These results suggest that the secondary structure of encapsulated insulin-loaded NPs was preserved after the encapsulation process.

### 3.4. Protein Recovery after Encapsulation Process

The percentage of insulin released from NPs and diffused from insulin-loaded PLGA NPs through the dialyzer was monitored along time.

**Figure 7:**
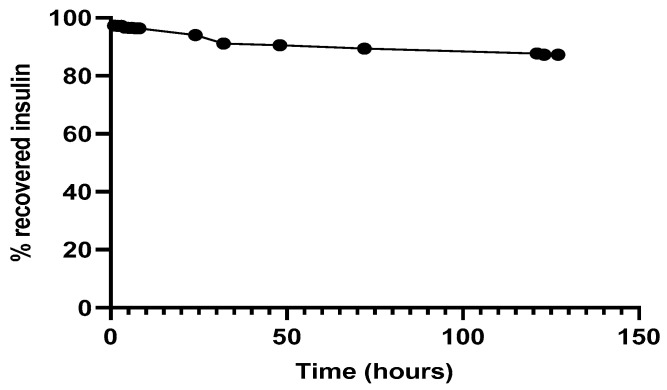
Recovery of insulin after released from NPs, 37 °C in PBS at pH 7.4.

Regarding the simultaneous insulin control assay, it was observed (Figure 7) that in the experimental conditions to which insulin had been submitted, there was practically no protein degradation, since the amount of insulin recovered and quantified, by the HPLC method previously described, was close to 100%. The retention time of 3.1 min remained unchanged.

#### In Vitro Release Assay

**Figure 8:**
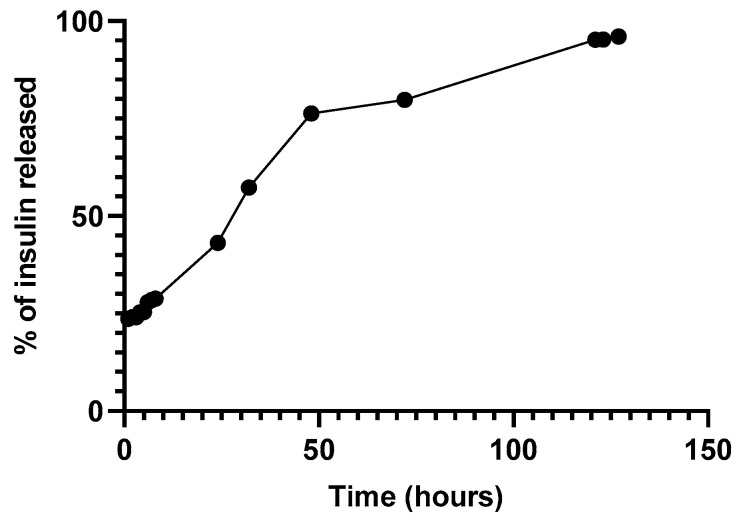
Release of insulin-loaded NPs through a Float-a-Lyzer at 37 °C in PBS pH 7.4.

Figure 8 depicts the release of insulin through a Float-a-Lyzer. The release profile suggested an initial bust phase after the first hours followed by a lag phase observed after 48 h [27,28].

**Figure 9:**
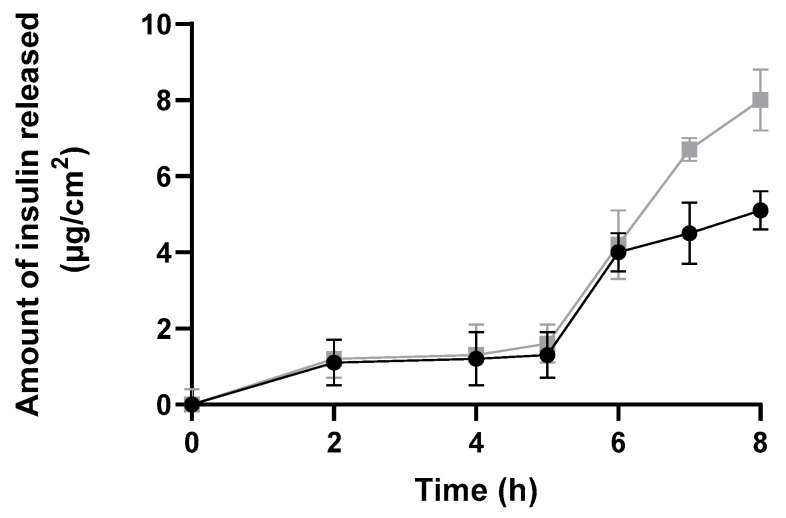
In vitro release profile through a silicone membrane from insulin-loaded NPs (black line), and a commercial solution of free insulin (grey line) over 8 h expressed in µg/cm^2^ (mean ± SD, n = 6).

The amount of insulin released and diffused from insulin-loaded NPs and commercial insulin solution through a silicone membrane was plotted against time over a period of 8 h to better evaluate the burst phase (Figure 9). This study showed a suitable and higher release profile (expressed as µg/cm^2^) for insulin-loaded NPs than for commercial insulin.

Both formulations presented a similar release profile, i.e., no significant amount of insulin was released up to 5 h. Then, the amount of insulin released increased in both formulations. Notwithstanding, it is important to highlight that the commercial insulin solution cannot be considered as a control solution, since it is not composed of the same solvents—neither the same insulin concentrations as insulin-loaded polymeric NPs. In fact, commercial insulin is four times more concentrated than NPs formulation. Therefore, both types of vehicles and their compositions varied, affecting the release profile.

### 3.5. Preliminary Safety and Efficacy Assessments

#### 3.5.1. In Vitro Safety Assessment

Cell viability was assessed by MTT assay. The tetrazole MTT, a yellow water-soluble compound, is converted to a purple, insoluble formazan product by mitochondrial dehydrogenases and reductase enzymes in active metabolic cells. Thus, the conversion of this product can be directly related to the number of viable (living) cells [29].

The effect in cell viability of commercial insulin in POLX gel, empty NPs in POLX gel, and insulin-loaded NPs in POLX gel (2.5 up to 80 µg/mL) was tested using a HaCaT cell line after exposure time for 12 h and 24 h (Table 2). It is possible to observe that HaCaT viability changed according to formulation concentration, formulation composition and time of exposure. The effect was dose and time-dependent.

This preliminary cell viability screening allows the determination of suitable exposure conditions in any cell-based assay, i.e., the incubation period of 12 h. This time point could be more accurate and realistic concerning the time of application and clearance of the product, and ultimately, the skin turn-over process.

#### 3.5.2. In Vivo Preliminary Efficacy Assessment

**Figure 10:**
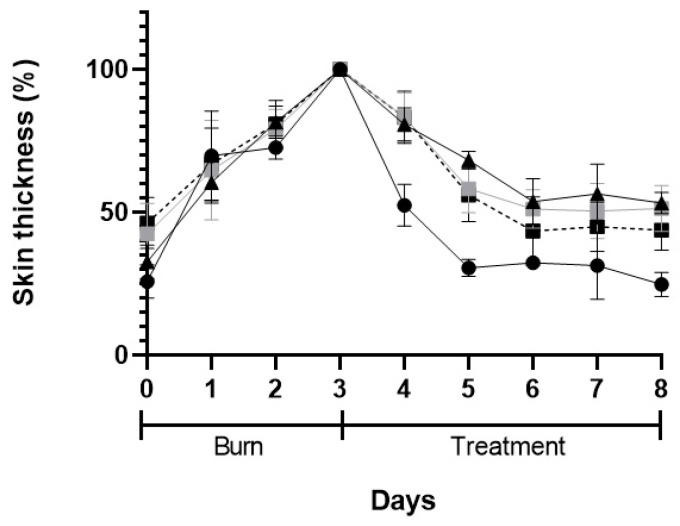
Skin thickness after skin burn chemically induced (3-days period) and during the 5-days of treatment: POLX gel (black triangles), commercial cream of silver sulfadiazine (black circles), insulin solution in POLX gel (grey squares), and insulin-loaded NPs in POLX gel (dark squares).

The macroscopic appearance of the wounds when POLX gel (negative control), commercial cream of silver sulfadiazine (positive control), insulin solution dispersed in POLX gel, and insulin-loaded NPs in POLX gel is presented on Table 3. No sign of infection was observed in any of the groups, except in the tested group dosed with negative control. The wound size reduction was relatively faster when insulin-loaded NPs were applied (based only on the macroscopic analysis). Analysis of the wound area showed a continuous increase in epithelialization with about 50% wound coverage at day 8, and complete wound closure by day 6 post wounding in the case of insulin-loaded NPs in POLX gel. The relative change of the skin thickness versus the initial value is represented in Figure 10. The thickness of the skin tends to increase with the injury and in the following days, due to the inflammation and subsequent skin regeneration with the formation of crusts. This increase was quite consistent across the different treatment groups. The test group had the same tendency as a positive control. An ideal result regarding the skin thickness level would be to obtain, at the end of the protocol, a skin thickness similar to the existent before the induction of the burn.

**Figure 11:**
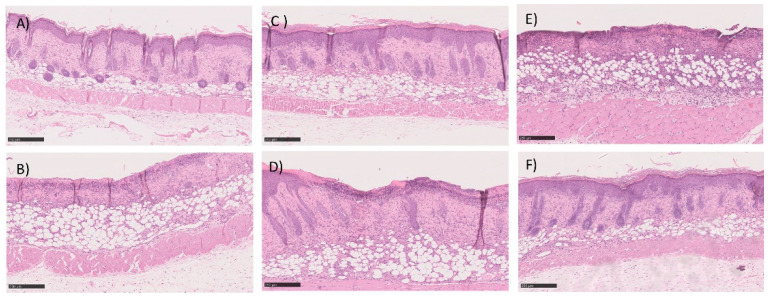
Histological section image of the skin of the mice after 5-days of treatment with (**A**,**B**) commercial cream of silver sulfadiazine, (**C**,**D**) commercial insulin in solution in POLX gel, and (**E**,**F**) insulin-loaded NPs in POLX gel. Scale bars 250 μm (magnification 100×).

The histopathological analysis is described in Table 4 and in Figure 11. Table 4 shows the classification of burns for each of the animals under study using a scoring system as previously described. The first point under analysis concerns the presence or absence of an ulcer. The second point assesses the degree of differentiation of the epidermis (absence or presence of granular layer in early or late stages, respectively). The amount of granulation tissue is classified in the third point. The next point refers to the inflammatory infiltrates. The last two points, the late stage of matrix remodeling, are associated with collagen, fiber orientation, and pattern. The last column of the table shows the sum of the score. A value of 16 corresponds to a fully regenerated lesion, while lower values mean that the regeneration is incomplete. The highest score values were obtained with the test group, but similar values were observed in all groups. Histological analysis also supported the absence of differences between all three groups. Briefly, the skin structure seems to be restored in most of the animals in both groups dosed with insulin and with commercial cream silver. The number of non-restored skin was also very similar between all groups. As a representative image, in these groups, Figure 11 depicted some examples of histological analysis of full, but some of the incomplete skin regeneration, supporting the score observed.

## 4. Discussion

As stated before, wound healing is a complex biological process that repairs damaged tissues and restores skin integrity. Insulin is described as a potent contributor to wound healing management [30]. Topical application of insulin for wound healing can be traced back to the 60 s and 70 s [31]. However, until the late 90 s the use of topical insulin to heal wounds decreased with only a few studies being performed. Recently, topical insulin has continuously garnered attention with the development of more advanced materials for the long-term release of bioactive insulin. In this study, insulin-loaded NPs were successfully prepared and characterized regarding their mean size, PdI, morphology, and encapsulation efficiency. According to the literature, particle size is a very important factor for skin penetration and permeation [32]. Passive penetration (without mechanical stress) into the skin of NPs higher than 300 nm does not seem to occur for human skin within 6 h after application [33,34]. Considering this, the insulin-loaded NPs prepared in this study should be retained in the burned area. On the other hand, insulin-loaded NPs were neutral in terms of surface charge favoring biocompatibility and cellular uptake [35]. AFM and SEM analyses showed a well-defined spherical shape of the formulated NPs. The spherical shape of the NPs is also important as it allows a greater contact surface with the damaged skin [36]. Finally, the neutral pH of the NP formulation showed good biocompatibility for topical application.

In the current study, more than half of the initial insulin added during the preparation method was encapsulated in NPs. The nature of each material seems to have a strong impact on this value. Insulin being hydrophilic it has a high affinity to the aqueous (external) phase, which increases the probability of diffusing out of the hydrophobic polymeric matrix. Finally, the stirring rate used during the addition of the coatings might have also caused some insulin loss, although without any impact on its biological activity.

Insulin remains stable after encapsulation. According to physical stability studies, this formulation was stable for at least 60 days, since both physical and chemical parameters remained constant, and no statistical differences were observed over time. In fact, preserving the protein stability after the encapsulation in NPs is a key requirement for any encapsulation process. Specifically, in this formulation, the risk of peptide degradation during NP production could be mainly due to the presence of organic solvents and shear rates used during the homogenization step that can lead to changes in the 3D structure of the protein, including alterations in the secondary structural elements. All analyses showed that insulin was completely recovered from NPs; insulin was stable and showed in the far-UV CD two negative CD bands around 208 and 223 nm, typical of a protein with high content of α-helices like insulin [37,38]. Thus, insulin encapsulated by this formulation method, i.e., modified-spontaneous emulsification solvent diffusion method, did not affect its secondary structure. Regardless of far-UV CD results deeming post-encapsulation insulin as being active; its activity was also confirmed in vivo, by analyzing the increased wound healing in mice after topical administration of the insulin nanoformulation.

In terms of release, an initial burst release after the first hours was followed by a lag phase: The first phase was supposed to be due to insulin diffusion through the external side of the polymeric matrix, and protein release during the matrix, or both [39,40]. The high hydrophobicity of the polymer contributes to a decreased degradation rate, and hence, a decrease in drug release rate [41,42].

Safety and efficacy are crucial factors for any formulation development. In this study, it was possible to observe that cell viability changed according to formulation concentration, composition, and exposure time and that the effect was dose and time-dependent. This fact could be due to several points. First, NPs could act as a physical barrier to the cells. This occlusive effect of NPs has been investigated by several groups; nevertheless, a very similar formulation made (with the same core) by our group showed that NPs were safe for the tested cells [43]. Thus, it should be another reason: all samples were tested in a Pluronic F-127 (POLX) gel to better mimic the potential use of insulin-loaded NPs in the further studies: topical use. POLX is a triblock copolymer, poly(ethylene oxide-*b*-propylene oxide-*b-*ethylene oxide) (PEO-*b*-PPO-*b*-PEO), consisting of PEO units (70%) and PPO units (30%). This polymer is converted from a low-viscosity aqueous solution to a physical network upon heating to body temperature at concentrations higher than 12% [44]. In the present study, we used a higher concentration of POLX (18%) and we hypothesized that the decrease of the cell viability may be attributed to the formation of gel at 37 °C (temperature of incubation of cells), causing a reduction in the amount of oxygen available to the cells [45]. It is also important to notice that commercial insulin has compounds other than preservatives that might have some influence on cell viability (an in vitro scenario).

Finally, an in vivo preliminary safety and efficacy assay was performed. In this assay, the aim was to reproduce a non-infectious wound model, but at the same time to reproduce a non-excisional or incisional. In these cases, the stratum corneum is defective, and the skin barrier is generally compromised. In this study, these preliminary results appear to be favorable regarding tissue regeneration after skin burn; since a good progression based on macroscopical analysis over five days was observed in animals treated with insulin-loaded NPs, very similar to positive control. Histological analysis also supports this similarity. When compared to free insulin, PLGA NPs probably delayed the release of insulin and might explain the observed differences in terms of the macroscopic evolution. The mechanism of insulin by itself in the wound healing process has already been described [10]. Insulin accelerates the growth of different cell types, affecting the proliferation, migration, and production of keratinocytes, endothelial cells, and fibroblasts [46]. However, according to the literature, the composition of PLGA in glycolic acid and lactic acids by itself is described to have an important role in the wound healing process. Specifically, in wound healing processes, hypoxia is transiently enhanced to coordinately rescue the injured tissue. Hypoxia, which in wounds originates from microcirculatory damage and increased oxygen consumption by inflammatory cells, promotes the glycolytic switch, a key metabolic adaptation contributing to cell preservation. A direct consequence is the release of lactic acid, the end-product of glycolysis. Lactate has been merely considered as a metabolic by-product. It is recognized as an important contributor to wound healing. Thomas K. Hunt and colleagues postulated that the increased concentration of lactate in wounds was a major signal for collagen synthesis and wound repair [47]. The underlying mechanism was shown to involve the activation of collagen prolylhydoxylase (PHD, an enzyme controlling procollagen hydroxylation and collagen maturation) in fibroblasts through a lactate-dependent decrease in PHD mono-ADP-ribosylation. Some other previous works support the role of PLGA in wound healing [48], for example, the application of exogenous lactate released from PLGA polymer accelerated angiogenesis and wound healing processes. These authors suggested an additive effect of lactic acid from PLGA and encapsulated curcumin for the active healing of wounds because of its innate lactic acid activity and sustained drug release [17]. Moreover, it is also known that PLGA has some other interesting properties in the wound healing process in terms of cell adhesion, and mechanical strength of wound dressing [49].

Currently, there was a concern about the shortening effect of the insulin-loaded NPs, and free insulin. This insulin effect in shortening the healing process was already seen in other works. For example, Apikoglu et al. (2010) showed that applying an insulin solution twice daily for 15 days enhances wound healing in diabetic and non-diabetic rats by shortening the time needed for complete epithelialization [10]. Most of the studies in the literature described insulin injections instead of non-invasive topical application, and some of them showed longer recovery time, higher insulin doses and a larger number of applications [50]. In the present study, one daily dose, a lower insulin dose, and a topical use (non-invasive application) were tested. Thus, several advantages are associated with this novel type of topical insulin formulation.

## 5. Conclusions

A stable insulin polymeric nanoformulation was successfully produced using PLGA. Post-encapsulation, insulin stability was assessed by different techniques, and all of them confirmed that insulin was completely released from NPs and its structure was preserved. In vitro and in vivo preliminary safety and efficacy assessments, respectively, showed very promising results, especially those regarding shortening the time of recovery of skin burns, very similar to the tested positive control. Taken together, as an alternative to conventional therapies, our studies support that topical insulin improves wound healing without causing side effects. Further studies are needed to improve our understanding the role of insulin plays in the healing of various types of wounds.

## Figures and Tables

**Table 1:** Characterization of insulin-loaded poly-DL-lactide/glycolide (PLGA) NPs and empty PLGA NPs. All data are presented as mean ± SD (at least n = 3). NPs means nanoparticles.

Formulations and Parameters	Size (nm)	PdI	Zeta Potential (mV)	EE (%)	pH
Empty NPs	223 ± 1	0.19 ± 0.01	−1.03 ± 0.26	-	5.5 ± 0.1
Insulin-loaded NPs	557 ± 19	0.38 ± 0.02	0.07 ± 0.06	55.3 ± 0.01	7.1 ± 0.2
Insulin-loaded NPs after solvent evaporation	520 ± 8	0.40 ± 0.02	0.25 ± 0.12	56.4 ± 0.03	8.7 ± 0.2

**Table 2:** HaCaT cell viability (%) of commercial (free insulin) in Pluronic ^®^ F127 (POLX) gel, empty NPs in POLX gel, and insulin-loaded NPs in POLX gel for 12 h and 24 h. All data are presented as mean ± SD.

Sample	Concentration (µg/mL)	% of Viable HaCat MTT 12 h	% of Viable HaCat MTT 24 h
Commercial insulin solution in POLX gel	2.5	100.0 ± 4.9	79.2 ± 7.2
	20	90.1 ± 5.4	65.3 ± 2.9
	80	71.5 ± 5.6	52.7 ± 7.1
Empty NPs in POLX gel	2.5	96.8 ± 9.8	56.8 ± 8.7
	20 80	87.2 ± 7.1 60.4 ± 3.6	55.2 ± 6.5 47.7 ± 4.8
Insulin-loaded NPs in POLX gel	2.5	93.3 ± 6.4	46.0 ± 6.1
	20 80	69.7 ± 4.6 56.2 ± 9.1	39.4 ± 5.6 36.0 ± 1.7

**Table 3:** Representative monitorization of the macroscopic appearance of the wounds on the 2nd, 5th, and 8th days after chemical burn induction.

Sample	Day 2	Day 5	Day 8
Negative control (POLX gel)	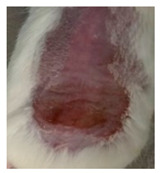	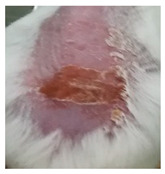	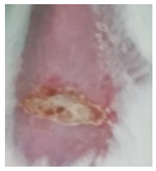
Positive control (Commercial cream of silver sulfadiazine)	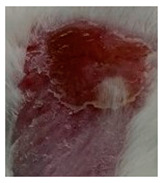	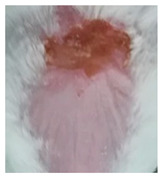	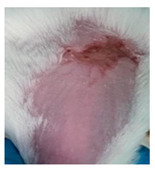
Commercial insulin solution in POLX gel	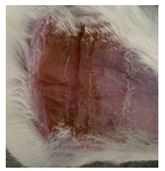	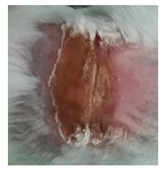	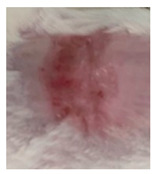
Insulin-loaded NPs in POLX gel	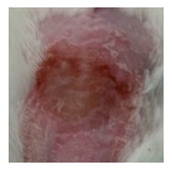	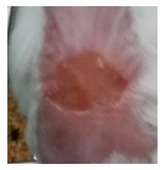	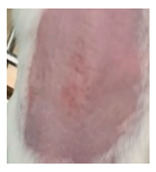

**Table 4:** Scoring of skin regeneration after 5-days of treatment (mean ± SEM), i.e., same end-point for all groups. Epidermal closure (ulcer 0 or closed 1); Epidermal differentiation (absent 0, spinous epidermal 1 or granular layer 2); Amount of granulation tissue (profound-1, moderate-2, scanty-3 or absent-4); Inflammatory infiltrate (plenty-1, moderate-2 or few-3); Collagen fiber orientation (vertical-1, mixed-2 or horizontal-3) and Pattern of collagen (reticular-1, mixed-2 or fascicle-3).

Samples	Epidermal Closure	Epidermal Differentiation	Amount of Granulation	Inflammatory Infiltration	Collagen Fiber Orientation	Pattern of Collagen	Total Score
Negative control (POLX gel)	0.5 ± 0.4	1.0 ± 0.7	3.0 ± 0.7	2.5 ± 0.4	2.5 ± 0.4	2.5 ± 0.4	12.0 ± 2.8
Positive control (Commercial cream of silver sulfadiazine)	0.7 ± 0.3	1.3 ± 0.5	3.3 ± 0.5	2.7 ± 0.3	2.3 ± 0.5	2.3 ± 0.5	12.7 ± 2.7
Commercial insulin solution in POLX gel	0.8 ± 0.2	1.5 ± 0.4	3.5 ± 0.4	2.3 ± 0.2	2.8 ± 0.2	2.8 ± 0.2	13.5 ± 1.6
Insulin-loaded NPs in POLX gel	0.8 ± 0.2	1.5 ± 0.4	3.5 ± 0.4	2.5 ± 0.3	2.8 ± 0.2	2.8 ± 0.2	13.8 ± 1.7

## Data Availability

The databases created and analyzed throughout the study are available upon request to the corresponding author.
